# Arrhythmogenic right ventricular cardiomyopathy type 6 (ARVC6): support for the locus assignment, narrowing of the critical region and mutation screening of three candidate genes

**DOI:** 10.1186/1471-2350-7-29

**Published:** 2006-03-28

**Authors:** Luzuko O Matolweni, Soraya Bardien, George Rebello, Ekow Oppon, Miroslav Munclinger, Rajkumar Ramesar, Hugh Watkins, Bongani M Mayosi

**Affiliations:** 1 The Cardiac Clinic, Department of Medicine, Groote Schuur Hospital and University of Cape Town, Cape Town, South Africa; 2 MRC/UCT Research Unit for Human Genetics, Division of Human Genetics, Department of Clinical Laboratory Sciences, Institute of Infectious Disease and Molecular Medicine, University of Cape Town, Cape Town, South Africa; 3 Sub-department of Cardiology, Department of Medicine, University of Kwa-Zulu Natal, Durban, South Africa; 4 Department of Cardiovascular Medicine, John Radcliffe Hospital, University of Oxford, Oxford, UK; 5 Centre for Human Genetics Research and Education, Faculty of Health Sciences, University of Stellenbosch, Tygerberg, Cape Town, South Africa; 6 Department of Medicine and Cardiology, Mafraq Hospital, Abu Dhabi, United Arab Emirates

## Abstract

**Background:**

Arrhythmogenic right ventricular cardiomyopathy (ARVC) is a heritable disorder characterized by progressive degeneration of right ventricular myocardium, arrhythmias and an increased risk of sudden death at a young age. By linkage analysis, ARVC type 6 was previously mapped to a 10.6 cM region on chromosome 10p12-p14 in a large North American kindred. To date, the genetic defect that causes ARVC6 has not been identified.

**Methods:**

We identified a South African family of 13 members with ARVC segregating as an autosomal dominant disorder. The diagnosis of ARVC was based on international diagnostic criteria. All available family members were genotyped with microsatellite markers at six known ARVC loci, and positional candidate gene screening was performed.

**Results:**

Genetic linkage and haplotype analysis provided lod scores that are highly suggestive of linkage to the ARVC6 locus on chromosome 10p12-p14, and the narrowing of the critical region to ~2.9 Mb. Two positional candidate genes (*ITG8 *and *FRMD4A*) were screened in which defects could possibly disrupt cell-cell adhesion. A non-positional candidate gene with apoptosis inducing properties, *LAMR1P6 *(laminin receptor 1 pseudogene 6) was also screened. Direct sequencing of DNA from affected individuals failed to detect disease-causing mutations in the exonic sequences of the three genes investigated.

**Conclusion:**

The narrowing of the ARVC6 critical region may facilitate progress towards the identification of the gene that is involved in ARVC. Identification of the causative genes for ARVC will contribute to the understanding of the pathogenesis and management of this poorly understood condition.

## Background

Arrhythmogenic right ventricular cardiomyopathy (ARVC, OMIM: 604401) is characterized by progressive degeneration and fibro-fatty replacement of right ventricular myocardium, causing ventricular tachyarrhythmias and resulting in sudden death, often during exercise. In Italy, where the condition has been studied extensively, ARVC accounts for 20% of all sudden deaths in individuals under 35 years and 22% of sudden deaths in athletes [1]. Familial occurrence is reported in 30–50% of individuals with ARVC, suggesting that genetic factors may play an aetiological role in a significant proportion of affected people [2]. At least 11 loci have potentially been identified so far: ARVC1 (14q23-q24), ARVC2 (1q42-q43), ARVC3 (14q12-q22), ARVC4 (2q32.1-q32.3), ARVC5 (3p23), ARVC6 (10p12-p14), chromosome 10q22.3, ARVD8 (6p24), ARVC9 (12p11), Naxos disease (17q21), and ARVC with anterior polar cataract [3,4]. To date five candidate genes have been identified, i.e., ryanodine receptor (*RyR2 *on 1q42), plakoglobin (*JUP *on 17q21), desmoplakin (*DSM *on 6p24), plakophilin-2 (*PKP2 *on 12p11), and transforming growth factor β3 gene (*TGFβ3 *on 14q24.3) [4,5]. Recently, a serendipitous mouse model of ARVC was discovered in which a functional laminin 1 receptor (*lamr1*) retroposon interacted with heterochromatin protein 1 (*HP1*) to cause degeneration of cardiomyocytes in the right ventricle of the heart [6].

Several lines of evidence suggest that impairment of cell-to-cell adhesion leading to accelerated apoptosis of myocardial cells may be the underlying pathogenic mechanism in ARVC. According to this hypothesis, disruption of cellular junctional cohesion leads to cell death and repair by fibrofatty replacement [7]. Initial support for this hypothesis was provided by the observation of abnormalities of myocyte intercalated discs on electron microscopy [8,9]. Subsequently, the involvement of desmosomal proteins (i.e., plakoglobin, desmoplakin and plakophilin-2) in different ARVC clinical phenotypes has been reported [7,10-12]. ARVC2, which has features that are more typical of a catecholamine-dependent ventricular tachycardia syndrome than ARVC, is associated with a mutation in ryanodine receptor-2 [13]. Mutations in the regulatory regions of the *TGFβ3 *have been found in association with one of the three families linked to the ARVC2 locus, and in one isolated case of ARVC [5]. The role of *TGFβ3 *mutations remain to be confirmed as a cause of ARVC because of the absence of mutations in the other two families that have been linked to this locus [4]. The difficulty in identifying the genetic mutations underlying ARVC2, which was initially mapped more than 10 years ago [14], underscore the challenge of cloning causal genes in rare disorders with small families, such as ARVC.

It is of interest that the discovery of the plakophilin-2 gene on chromosome 12p11 as a cause of ARVC has resulted in the re-assignment of the disease locus in one small European family. Originally, this family was thought to be linked to chromosome 2q32.1-q33.3 using a cumulative lod score together with two other small families [15]. Individually, these families did not produce statistically significant lod scores. Subsequently, a frame shift mutation was identified in the plakophilin-2 gene in the European family, which shows that the original linkage findings to chromosome 2 in this family were erroneous [12].

Therefore, independent corroboration of genetic linkage findings, particularly in rare conditions such as ARVC, is essential to facilitate progress towards the identification of causal genetic mutations. We report on the linkage analysis in a South African family with ARVC, which provided support for previous chromosomal localization of ARVC6, the narrowing of the critical region, and exclusion of disease-causing mutations in the coding regions of two positional and one non-positional candidate genes.

## Methods

### Family and clinical evaluation

A family with ARVC was investigated at Wentworth Hospital, Durban and Groote Schuur Hospital, Cape Town, South Africa. Family members gave informed consent to participate in the study, which was approved by the local ethics committee. The protocol included: 12-lead electrocardiogram (ECG), signal averaged ECG, 24-hour ambulatory ECG, and 2D echocardiogram. Additional procedures (i.e., cardiac magnetic resonance imaging, right ventricular angiogram, and endomyocardial biopsy) were performed when clinically indicated. The diagnosis in the index case was based on criteria proposed by McKenna et al [16]: i.e., affection status was established when two major criteria, or one major criterion plus two minor criteria, or four minor criteria were fulfilled. The diagnosis of ARVC in the other family members was based on the criteria for familial ARVC proposed by Hamid et al [2]; i.e., the presence of one or more minor criteria in the context of familial ARVC indicate an affected status.

### Molecular genetic studies

Peripheral blood samples were collected from family members in EDTA-coated tubes and genomic DNA was prepared from nucleated blood cells, according to the salting out method. PCR-based methods were used for genotyping and sequencing. All PCR amplifications were carried out on a GeneAmp 9700 thermocycler (Applied Biosystems). Each 50 μl reaction contained 200 ng of genomic DNA, 200 μM dNTPs, 50 mM KCL, 10 mM Tris (pH 8.4), 10 pM of each primer and 1 unit of BIOTAQ DNA polymerase (BIOLINE). The concentration of magnesium chloride was varied depending on the GC content of the primers used. The PCR protocol required an initial denaturation step of 2 minutes at 95°C, followed by 25 cycles of denaturation at the same temperature, annealing at different temperatures depending on primer characteristics, and extension at 72°C for 1 minute. A final extension step for 2 minutes was included. The duration and temperature profiles for the annealing cycle segments were altered depending on the primer sequence and type of application (e.g., genotyping or sequencing). For visualization of the PCR product in non automated and automated systems, ethidium bromide or fluorescently-labeled primers were used, respectively. Both the ABI PRISM model 377 and 3100 genetic analyzers from Applied Biosystems were used in the genotyping and sequencing experiments.

### Linkage analysis and the critical region

PCR primer sequences for the different genetic markers were obtained from the Genome Database [17]. The following markers were used to assess linkage to the following ARVD loci: ARVD1 – D14S42, D14S999; ARVD2 – D1S2925, D1S180; ARVD3 – D14S252, D14S80; ARVD4 – D2S364, D2S389, ARVD5 – D3S1259, D3S1293, and ARVD6 – D10S464, D10S547, D10S1705, D10S2325, D10S1707, D10S1664, D10S191, D10S1653, D10S1477, D10S1661, D10S504, D10S2318, D10S548, D10S466, D10S245. Two point lod scores were calculated using the MLINK program of the LINKAGE package (version 5.10). The program parameters were set for that of an autosomal dominant inherited disorder at two levels of penetrance: 60% and 95%. The allele frequencies for the normal and disease alleles were set at 0.9999 and 0.0001, respectively. Haplotypes in the pedigree were constructed using the Genehunter program [version 2.0 beta (r2)]. Genetic distances (in centiMorgans) between markers and the order of the markers, which were specified for the construction of haplotypes, were determined from the Marshfield [18], the University of California Santa Cruz [19], and LocusLink [20] databases.

### Bioinformatic analysis of the ARVD 6 critical region

The genomic sequence for the ARVD6 critical region between markers D10S1707 and D10S1477 was downloaded from the UCSC Genome Browser [assembly: Human Nov 2002]. A comparative analysis of the sequence with that from the National Center for Biotechnology Information (NCBI) non-redundant database was performed using the option *blastn *[21] to detect possible contaminating sequences from other chromosomes. LocusLink was used to determine the gene structure of *ITGA8 *and *FRMD4A *candidate genes, whereas the Vertebrate Genome Annotation database was used for the *LAMR1P6 *pseudogene. The genes in the critical region were positioned relative to the microsatellite markers. Information about the expression and putative gene function of the genes was obtained from various databases linked to the University of California Santa Cruz genome database [19], LocusLink [22], Gene Ontology [22], Gene Cards [23], and Stanford SOURCE [24].

### Mutation screening of the candidate genes

The majority of the genes (e.g., desmoplakin, plakoglobin and plakophilin-2) that cause ARVC code for proteins that are involved in cell-cell adhesion. For this reason, genes that are involved or have a potential to play a role in cell-to-cell adhesion were prioritized for mutation screening. Annotated genomic sequence of each of the candidate genes was downloaded from the NCBI database in Genebank format and a perl script written by one of us (GR) was used to determine the start codon, intron-exon boundaries, branch sites and the stop codons. PCR primers flanking the exons of each of the candidate genes were designed to amplify at least 30 bases on either end of the exon to cover the splice site junctions. The PCR primers used in this study are provided in Table 1. Direct sequencing of both strands was performed, and each exon was amplified from genomic DNA of affected and unaffected individuals, purified using the QIAGEN gel purification system and sequenced using the BIG DYE V3.1 dideoxy-terminator kit (Applied Biosystems). Sequence analysis (Applied Biosystems 2001), Seqscape (Applied Biosystems 2002) and Bioedit [25] software programs were used to edit and assemble the sequences. Where a variant was identified in only the affected individual, mutation screening of the entire family for the variant was performed to determine whether the variant was segregating with the disease. If the sequence variant was found to be tracking with the disease, the frequency of its occurrence in the background population was determined. All sequence variants occurring at a frequency of ≥ 1 % in the background population were regarded as polymorphisms.

**Table 1:** Oligonucleotide primers used for PCR amplification and DNA sequencing of the exons of the *ITGA8, FRMD4A, and LAMR1P6 *genes.

Gene	Exon No.	PCR fragment size (bp)	Forward primer (5' → 3')	Reverse primer (5' → 3')	Melting temperature (°C)
					
*ITGA8*	1	587	acc gcc aga ttc cac cag	tgggtc ttc tgg gtc ctg t	58
	2	300	ggg cag gag gtt aca gag ac	acc aag aca gcg gga agt c	56
	3	300	gag gag ttt gac aag gat tgg	gct ttc cta aac atg agc ttg	54
	4	368	ggc caa tta att cac cca at	tac cta ccc ccg aag ctt tt	48
	5	399	tca caa cgc tat ttg gtg taa ga	cag gct agg tat gtt cct caa ga	58
	6	223	tct ctc aag gga gag agt tca a	tgt ttg agg tga tgg tta tgc t	45
	7	296	ttt caa ggg aat gtg ggt ct	cat cgg ata agt cag gga ta	54
	8	249	tgg tag ccc ttt cac ctt tt	ctc cat taa tat ccc ttt gg	56
	9	250	ata tgg cgt gtg tgt gtg tg	cgg tgg att agg tgt att aag tg	54
	10	238	cca att cta tgg caa atg gt	tgc cta gga cag ttt cca gt	56
	11	329	att cac tgg gtc agg ggt tag	tga ggc aat aag gaa ggg tgt	58
	12	464	taa caa agc agc gaa agc aa	taa ctg cac cca cac aca cc	58
	13	475	tag aga agt tga ggc agg agg atc act tg	gct tca gcc tca tct gta gaa gaa aac tgc	56
	14	250	acc atc agc atg tag ggg ta	ccc cag aat aaa tcg ttg ga	55
	15	243	Atg cag tga agt ggg ctt ct	caa gat acc cag gtg atg tga a	55
	16	553	ttg gta gaa ggg gtg tgt g	gca tga taa atg agc tgg aa	56
	17	468	ttc cag ctc att tat cat gc	gaa gtc agg gtc aag agg ag	55
	18	399	aca taa atc agc ctc cat cta agc	cat gca gaa gta aaa atg aaa gga	56
	19	278	ttt gtg gca cca ttc aga att t	caa gaa ttt cac gca gca tag tt	56
	20	460	gat gat tct ctt gcc tct tcc tac	gtg gct cat tgc tgt tat gat tta	56
	21	299	ctc ctg ggt gtt tga gat cc	agg tgc tga gtt gct gag tg	58
	22	244	ggg ttt tgc agg aat gac tt	aag ggc ctt gac aga tga c	58
	23	268	cgt gtg ttc tcc tgt gat atg	cat cag gaa aat tcc aca tca	55
	24	324	gaa aac cac ttt ctt ctg ctt c	cac agc gag atc ctg tct ca	56
	25	396	tgc cat atc gac ttt gaa tt	aat caa caa agc cac tga	55
	26	369	cac agg tct gga tcc tca tgt	tga gca ctg aag gag acc aa	58
	27	298	ggt gat gac ttt tga gga aat ga	cgc tat gaa aag act tcc cac t	58
	28	263	att agg tgg ctg cca gta taa c	acc act aaa gcc tag cac aag c	56
	29	380	gga tcc ttt cag atc aac ttc c	gca aac aag aca cga ttt tcc	56
	30	365	ctg ggg aat aga gag cga gac	gtg cgg tgt aga tga ggt gat	58
					
*FRMD4A*	1	597	acc tcc tat gcc tcc ctg aa	ctg gca gca att ctt cct tt	58
	2	371	cac agt tcc cac cct gtc tt	aaa gag cct gcg gga taa ag	58
	3	395	ata cag tgc cga ctc cca ga	gga tgc ctt agg ctg gtg ta	58
	4	387	gac cct tga ctt ggc tgt gt	caa gtg gtc tgc agc tga tt	58
					
*FRMD4A *splice variant	1	688	ggt gag cat gtg acc tac tgt	cca act gtt cag gaa acg tg	58
	nested primers		gc gtg act tgc atg atg tga c	ccc aag cct tat agc act aaa c	
					
*LAMR1P6*	1	884	ttc cca tcg caa ctt aaa gg	caa cgt tgt ttc cat gtt gc	58
	nested primers		aca gag caa tgg tgg gaa ag	ctt tcc cac cat tgc tct gt	
	nested primers		aat tgc tgg cca ctt cac tc	caa ctg cat caa acc cac tg	

## Results

### Clinical findings

**Figure 1:**
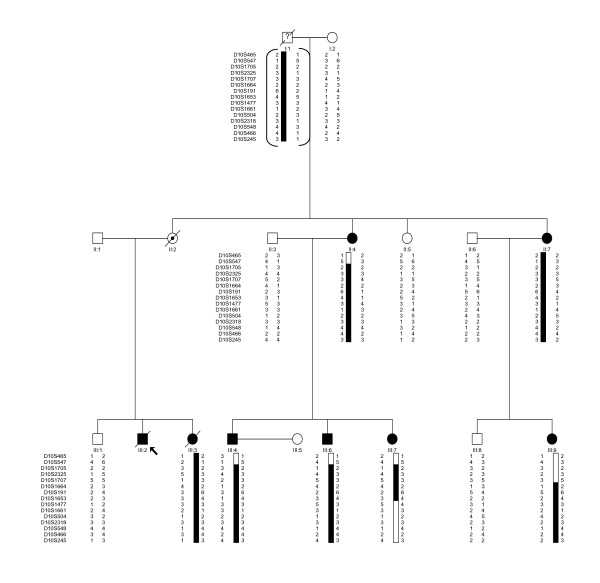
Pedigree of the ARVC family studied, showing pedigree numbers by generation and person number (i.e., I:1 to III:9), and genotypes useful for delimiting crossover points. Markers names at the left of each generation. Individuals III:7 and III:9 have recombinations that delimit the region. The disease-associated haplotype is shaded in black. Genotypes for individual I:1 are inferred.

We identified a 3-generation family of northern European descent from the KwaZulu-Natal province of South Africa. Thirteen members were available for clinical and genetic study, seven of whom were clinically affected. Clinical features of the affected individuals are summarized on Table 2 and the pedigree is presented in Figure 1. The index case (III:2) was a young male who, at 16 years, presented with palpitations associated with ventricular tachycardia that had left bundle branch morphology in keeping with a right ventricular origin. His resting 12-lead electrocardiogram (ECG) showed an epsilon wave in lead V1 and inverted T waves in V1-3. The echocardiogram and right ventriculogram demonstrated a markedly dilated right ventricle with reduced systolic function. He died suddenly at the age of 22 years despite treatment with various anti-arrhythmic drugs. His sister (III:3), who had similar clinical features, died suddenly at the age of 24 years. No clinical information was available for their mother (II:2) who died of brain cancer at the age of 34 years, and their father (II:1) was unavailable for study. The grandfather (I:1), who is likely to be the gene carrier, died suddenly of suspected coronary thrombosis at the age of 52, and the maternal grandmother of the index case (I:2) is unaffected.

**Table 2:** Clinical features of affected members with familial arrhythmogenic right ventricular cardiomyopathy.

Subject	Age at diagnosis (yrs.)	Sex	Clinical presentation	ECG and EPS abnormalities	RV abnormalities on imaging studies	Pathological findings on endomyocardial biopsy or autopsy	Major criteria	Minor criteria
II:4	44	F	Asymptomatic	T wave inversion V1-3	None found on echo MRI not done	Not performed	0	2
II:7	44	F	Palpitations	T wave inversion V1-3	Mild right ventricular dilation with wall motion change and aneurismal abnormality on MRI	Not performed	0	3
III:2	16	M	Palpitations	T wave inversion V1-3 Epsilon wave, V1 VT with LBBB morphology Inducible VT	Dilated RV with hypokinetic outflow tract	Not performed	2	2
III:3	13	F	Palpitations	VT with LBBB morphology	Dilated RV with hypokinesia	Not performed	1	2
III:4	23	M	Palpitations, chest pain	T-wave inversion V1-5 PVCs with LBBB pattern	Dilated RV on echo	Fibrofatty replacement of RV myocardium on endomyocardial biopsy	1	4
III:6	21	M	Asymptomatic		Dilated RV on echo; Evidence of fatty infiltration of RV wall on MRI	Not performed	1	2
III:7	19	F	Syncope	Frequent PVCs (>1000/24hrs)	Dilated RV on echo and MRI; Fatty infiltration of RV wall on MRI	Not performed	1	4
III:9	16	F	Asymptomatic	None	Mild right ventricular dilation with wall motion change and aneurismal abnormality on MRI	Not performed	0	2

Note: Echo, echocardiography; ECG, electrocardiogram; EPS, electrophysiological study; F, female; LBBB, left bundle branch block; M, male; MRI, magnetic resonance imaging; RV, right ventricle; VT, ventricular tachycardia; PVCs, premature ventricular complexes.

A cousin of the index case (III:4) presented with chest pain and palpitations at the age of 23 years. He had frequent ventricular ectopy of >1000/24 hours on 24 hour ECG (Holter) monitoring, global dilatation of the right ventricle on echocardiography and angiography, changes suggestive of increased amount of fat in the right ventricular free wall with complete replacement in areas on cardiac magnetic resonance imaging, which was confirmed on histological examination of the right ventricle following endomyocardial biopsy. At electrophysiological study, inducible non-sustained monomorphic ventricular tachycardia with a left bundle branch morphology and right axis deviation was detected. The siblings of III:4 (III:6 and III:7) were found during family screening to have structural and ECG abnormalities consistent with a diagnosis of familial ARVC [2,26]. The mother (II:4) of subjects III:4, III:6, and III:7 only had an abnormal ECG showing T wave inversion in the anterior leads (V1-3), which in the context of familial disease, is compatible with a diagnosis of ARVC with partial penetrance [2,26]. Similarly, individual II:7 had palpitations, T wave inversion in the anterior leads (V1-3), and mild right ventricular dilatation, aneurysmal change and wall motion abnormality on cardiac magnetic resonance imaging. Her daughter, who was examined at age 16 (III:9) had similar cardiac magnetic resonance imaging abnormalities, but the son (III:8) was clinically unaffected. The pedigree indicates autosomal dominant inheritance of ARVC with variable penetrance (Fig. 1).

### Linkage analysis

The known ARVC loci on chromosomes 14q23-q24 (ARVC1), 1q42-q43 (ARVC2), 14q12-q22 (ARVC3), 2q32.1-q32.3 (ARVC4), and 3p23 (ARVC5) were excluded as disease loci in this family (data not shown). Evidence for linkage was, however, detected when markers on chromosome 10p12-p14 situated close to the ARVD6 locus were investigated. This locus had previously been identified in a North American family displaying ARVC as an autosomal dominant trait with complete penetrance and an early age at onset [27].

The pedigree of the family is shown in Figure 1, along with haplotypes corresponding to part of the chromosomal region 10p12-p14. Two point lod scores for genotyping of 15 markers spanning the ARVD6 locus produced lod scores highly suggestive of linkage in this family (D10S191 and D10S1653; Z = 2.93 and 2.93 at θ = 0.00, respectively, penetrance 95%; Table 3). Lod score simulation analysis showed that 2.93 is the highest lod score that could be obtained for this pedigree. It is remarkable that the peak lod score was found at identical markers (D10S191 and D10S1653) to the original family in which the locus was initially described, which further supports the external validity of our result. It was, however, not possible to establish whether the North American [27] and South African families shared similar haplotypes because of differences in the labelling of alleles of the markers in the two studies.

**Table 3:** Two-point lod scores obtained for 15 markers on chromosome 10 in the South African family with recurrence of arrhythmogenic right ventricular cardiomyopathy. Lod scores were calculated for different values of penetrance (a: 60%; b: 95%)

		Recombination fraction						
Marker	Penetrance	0.00	0.01	0.05	0.10	0.20	0.30	0.40

D10S465	a	-2.87	-0.94	-0.31	-0.10	0.02	0.01	-0.02
	b	-3.94	-1.18	-0.54	-0.32	-0.17	-0.13	-0.09
D10S547	a	-6.39	-1.60	-0.34	0.09	0.34	0.31	0.17
	b	-8.67	-1.11	0.13	0.53	0.70	0.59	0.34
D10S1705	a	0.02	0.02	0.02	0.02	0.02	0.01	0.01
	b	-0.11	-0.11	-0.08	-0.06	-0.01	0.01	0.01
D10S2325	a	0.91	0.89	0.80	0.68	0.43	0.21	0.05
	b	1.16	1.14	1.03	0.89	0.60	0.31	0.08
D10S1707	a	1.33	1.31	1.22	1.09	0.83	0.54	0.24
	b	1.33	1.32	1.26	1.18	0.96	0.69	0.37
D10S1664	a	0.59	0.57	0.51	0.43	0.29	0.16	0.06
	b	0.70	0.68	0.63	0.56	0.42	0.28	0.13
D10S191	a	2.43	2.38	2.21	1.98	1.49	0.95	0.40
	b	2.93	2.88	2.68	2.43	1.88	1.26	0.61
D10S1653	a	2.43	2.38	2.21	1.98	1.49	0.95	0.40
	b	2.93	2.88	2.68	2.43	1.88	1.26	0.61
D10S1477	a	-2.27	-0.20	0.39	0.55	0.55	0.40	0.19
	b	-3.06	-0.07	0.56	0.75	0.77	0.61	0.35
D10S1661	a	-1.88	0.19	0.73	0.84	0.73	0.48	0.20
	b	-2.28	0.68	1.21	1.28	1.11	0.77	0.37
D10S504	a	0.93	0.91	0.82	0.71	0.47	0.25	0.08
	b	1.22	1.20	1.10	0.98	0.72	0.44	0.18
D10S2318	a	0.55	0.54	0.48	0.41	0.26	0.13	0.03
	b	0.77	0.75	0.68	0.59	0.41	0.22	0.07
D10S548	a	1.0	0.98	0.89	0.77	0.53	0.29	0.10
	b	1.29	1.26	1.17	1.05	0.79	0.49	0.21
D10S466	a	-2.60	-0.57	0.02	0.20	0.24	0.15	0.06
	b	-3.38	-0.41	0.19	0.37	0.40	0.29	0.12
D10S245	a	0.55	0.54	0.48	0.41	0.26	0.14	0.04
	b	0.84	0.82	0.75	0.67	0.48	0.29	0.11

The analysis of recombination events in informative meioses for the 15 markers that were genotyped in the family facilitated the fine mapping of the locus. Construction of haplotypes revealed that individual III:7 (who is affected; Table 2) is recombinant for the markers D10S1477 through to D10S245 (Figure 1). In this individual the maternally inherited chromosome had recombined. This recombination event places the disease locus distal to marker D10S1477. Similarly, individual III:9, who is affected (Table 2) was found to be recombinant for markers D10S465 to D10S1707 (Figure 1). In this individual the recombined chromosome was inherited from her mother, II:7 who is clinically affected. These data place the disease locus proximal to marker D10S1707. Therefore, the critical region of the ARVD6 locus lies between markers D10S1707 and D10S1477. The typing of three additional markers within the new region, D10S1664, D10S191 and D10S1653 did not reveal additional recombination events.

The genomic sequence between markers D10S1707 and D10S1477 was found to span a region of ~2.9 Mb and was shown not to contain any contaminating sequence from other chromosomes using bioinformatics analysis. The observed ~2.9 Mb region marked a significant reduction from ~5.4 Mb that was previously reported in the North American family [27]. This reduction also significantly reduced the number of possible positional candidate genes in the region.

### Bioinformatic analysis

The critical region was found to contain 21 known (protein coding) genes. Evaluation of each candidate gene for expression and function revealed two candidate genes with cell-to-cell adhesion properties, integrin alpha 8 (*ITGA8*) and FERM domain containing 4A (*FRMD4A*) (Table 4). The *ITGA8 *gene is flanked by markers D10S1477 and D10S191, whereas marker D10S1653 is intragenic; markers D10S191 and D10S1653 had the highest lod scores in the study. The marker D10S1707, which is at the distal boundary of the ARVD6 locus, resides within intron 4 of the *FRMD4A *gene. Analysis of the gene structure of the candidate genes demonstrated that *ITGA8 *is comprised of 30 exons, while *FRMD4A *is comprised of 25 exons and a splice variant with 14 exons. Only exons 1 to 4 of *FRMD4A *that lie within the critical region were screened for mutations. Exon 1 of *FRMD4A *was unique to the splice variant and had a variable number of tandem repeats (VNTRs = 27 CA repeats) situated approximately 10 base pairs 5' to the start codon. Further investigation of the microsatellite by genotyping in affected and unaffected individuals did not show any significant expansion or reduction of the repeats, which could be considered as a possible disease causing mechanism.

**Table 4:** General information on candidate genes selected for mutation screening.

Gene symbol	Genomic size	No. of exons	Flanking markers	Function and Expression	Variants detected
*ITGA8*	0.20 Mb	30	D10S1477, D10S191	The product of this gene is a cell surface glycoprotein involved in cell-to-cell adhesion. ITGA8 plays a major role in the integrin-mediated signalling pathway. It is expressed in skeletal muscle, fetal heart, and liposarcoma.	133C>A base change in exon 17 resulting in Q276P amino acid change. Two intronic changes: IVS13 -38C>T and IVS18 +40A>G
*FRMD4A*	0.69 Mb	25	D10S1664, D10S2325	This gene encodes for a hypothetical cytoskeletal protein binding molecule that is concentrated in the undercoat of the cell-to-cell adherens junction and has similarity to the ERM proteins which are plasma membrane-actin filament cross linkers. Expression is increased in hepatic adenoma and squamous epithelium.	CA variable number tandem repeat (VNTR) polymorphism in exon 1 of the splice variant.
*LAMR1P6*	0.884 Kb	1	D10S600, D10S509	There is no information currently available on expression and function of this pseudogene.	346G>A base change.

Note: Echo, echocardiography; ECG, electrocardiogram; EPS, electrophysiological study; F, female; LBBB, left bundle branch block; M, male; MRI, magnetic resonance imaging; RV, right ventricle; VT, ventricular tachycardia; PVCs, premature ventricular complexes.

The *LAMR1P6 *pseudogene/retroposon (XM_053952) is a processed transposable element that is reported to occur within the ARVC6 critical interval [6]. However, the localisation of *LAMR1P6 *pseudogene/retroposon in our interval could not be verified, and has been placed 11.9 Mb outside the ARVC6 critical region (Celera Genome Map). The recent finding of a functional *lamr1 *retroposon as a cause of ARVC in the mouse and the possibility that an expressed pseudogene may regulate the messenger RNA stability of a homologous gene [28] suggest that the *LAMR1P6 *pseudogene/retroposon is a plausible candidate for ARVC in man. The fact that the related functional homologue *LAMR1P6 *is involved in apoptosis, which is thought to be an important pathogenic mechanism of ARVC, led us to screen for the *LAMR1P6 *pseudogene on chromosome 10p for mutations in this study.

### Mutation analysis

Data on the three candidate genes and all the variants detected in this study are summarised in Table 4. Direct sequencing of the candidate genes did not reveal any pathogenic mutations in neither the coding nor intron-exon junctions of affected individuals. A single nucleotide polymorphism (SNP) Q276P in *ITGA8*, was detected in the coding sequence of exon 17, and was found to co-segregate with the disease-associated haplotype in the family. Further investigation revealed that this SNP was present in 6 out of 314 chromosomes from the normal population, suggesting that it is a polymorphism. Mutation screening of the *LAMR1P6 *pseudogene revealed a single nucleotide polymorphism (346G>A) that was present in both affected and non-affected members of the family.

## Discussion

We report a South African family with ARVC segregating as an autosomal dominant trait for which we obtained lod scores highly suggestive of linkage to the ARVC6 locus on chromosome 10p12-p14 [27]. Unlike the original family in which linkage had been initially reported who demonstrated complete penetrance of the disease at an early age, we found variable expression of the disease with a tendency for severe manifestations in the third generation. In addition, we have defined crossover events that have significantly reduced the chromosomal region harbouring the disease gene to a 2.9 Mb interval between markers D10S1707 and D10S1477.

The refined chromosomal region of interest on chromosome 10p contains several candidate genes that we are presently evaluating. The vimentin gene, which had been considered previously as a good candidate [27] is no longer a positional candidate for the ARVD6 locus as defined by our haplotyping data. We screened the positional candidate genes, *ITGA8 *and *FRMD4A*, and the *LAMR1P6 *pseudogene since these genes are known to play a role in mediating either cell-extracellular matrix and cell-cell interactions or apoptosis. *ITGA8 *belongs to the integrin alpha chain family, and is a receptor for fibronectin and cytostatin, and recognizes the sequence R-G-D in its ligands. *FRMD4A *is highly concentrated in the undercoat of the cell-to-cell adherens junction and has similarity to the ERM proteins which are plasma membrane-actin filament cross linkers [29]. LAMR1 (also known as RPSA) is a ribosomal protein found in the nucleus and is involved in apoptosis [6]. The *LAMR1P6 *pseudogene may possibly play an important role in regulating the translational process by ensuring the stability of the messenger RNA of the wildtype *LAMR1 *gene. Such a mechanism has been characterized in mice whereby reduced transcriptional levels of makorin1-p1 result in reduced shelf-life of its homologue, makorin1 [28].

Mutation screening of the coding regions and intron-exon boundaries of *ITGA8*, *FRMD4A *and *LAMR1P6 *revealed only non-pathogenic changes. *LAMR1P6 *was excluded as being responsible for the disorder in this kindred as the only sequence variant detected was found in both affected and unaffected individuals. The presence of mutations in the regulatory regions of *ITGA8 *and *FRMD4A *genes, as has been reported in the case of ARVC1 and the *TGFβ3 *gene [5], cannot be ruled out.

The lod scores obtained in the current study did not reach the statistical threshold of significance of 3. However, the fact that this locus has previously been linked to ARVC with a lod score over 3, at the same markers (D10S191 and D10S1653; [27]) for which we have obtained our highest lod scores, supports the assertion of genetic linkage in this South African family. Additionally, the combined evidence provided in this manuscript of a disease-associated haplotype spanning 15 markers on chromosome 10p, as well as the fact that the maximum lod score that could be achieved for this family was obtained for two markers, is highly suggestive of linkage in this family.

The narrowing of the ARVD6 critical interval has improved the prospects for the identification of the causal gene for ARVC6. Once the gene and mutation are identified, it is possible that improved therapeutic options based on the underlying cause of disease may be developed, resulting in better long term care and survival for patients with ARVC and their family members who are at risk.

## Conclusion

We report a new family of northern European descent with ARVC segregating as an autosomal dominant trait that is potentially linked to the ARVD6 locus on chromosome 10p12-14. As this condition is rare with only a few relatively small families with ARVC reported around the world, it is important to provide independent corroboration for the ARVC loci. Reduction of the ARVC6 critical interval will improve the feasibility of the identification of the disease causing gene.

## Abbreviations

ARVC – arrhythmogenic right ventricular cardiomyopathy

ECG – electrocardiogram

FRMD4A – FERM domain containing 4

ITG8 – integrin alpha 8

LAMR1P6 – laminin receptor 1 pseudogene 6

MRI – magnetic resonance imaging

## Competing interests

Financial competing interests

• In the past five years we have not received reimbursements, fees, funding, or salary from any organization that may in any way gain or lose financially from the publication of this manuscript, either now or in the future.

• We do not hold any stocks or shares in an organization that may in any way gain or lose financially from the publication of this manuscript, either now or in the future.

• We do not hold nor are we currently applying for any patents relating to the content of the manuscript. We have not received reimbursements, fees, funding, or salary from an organization that holds or has applied for patents relating to the content of the manuscript.

• We do not have any other financial competing interests.

Non-financial competing interests

We have no non-financial competing interests (political, personal, religious, academic, intellectual, commercial or any other) to declare in relation to this manuscript.

## Authors' contributions

MM identified the family. BMM conceived the genetic study, performed the initial linkage analysis for markers for ARVC1-3 under the supervision of HW, and directed the subsequent research work. GR and EO performed the bioinformatics analysis and contributed to the experimental design of the work. MM and BMM conducted the phenotyping of the family members. HW and RR contributed to the experimental design. LOM, assisted by SB, conducted the genotyping of the family members; SB conducted the linkage analysis. All authors contributed to the writing of the manuscript, and they approve of the final draft that has been submitted for publication.

## Pre-publication history

The pre-publication history for this paper can be accessed here:


